# Evaluating the Clinical Impact of a Genomic Classifier in Prostate Cancer Using Individualized Decision Analysis

**DOI:** 10.1371/journal.pone.0116866

**Published:** 2015-04-02

**Authors:** Jennifer Mason Lobo, Adam P. Dicker, Christine Buerki, Elai Daviconi, R. Jeffrey Karnes, Robert B. Jenkins, Nirav Patel, Robert B. Den, Timothy N. Showalter

**Affiliations:** 1 Department of Public Health Sciences, University of Virginia School of Medicine, Charlottesville, VA, United States of America; 2 Department of Radiation Oncology, Jefferson Medical College and Kimmel Cancer Center, Thomas Jefferson University, Philadelphia, PA, United States of America; 3 GenomeDx Biosciences, Vancouver, British Columbia, Canada; 4 Department of Urology, Mayo Clinic, Rochester, MN, United States of America; 5 Department of Laboratory Medicine and Pathology, Mayo Clinic, Rochester, MN, United States of America; 6 Department of Radiation Oncology, University of Virginia School of Medicine, Charlottesville, VA, United States of America; University of Kentucky College of Medicine, UNITED STATES

## Abstract

**Background:**

Currently there is controversy surrounding the optimal way to treat patients with prostate cancer in the post-prostatectomy setting. Adjuvant therapies carry possible benefits of improved curative results, but there is uncertainty in which patients should receive adjuvant therapy. There are concerns about giving toxicity to a whole population for the benefit of only a subset. We hypothesized that making post-prostatectomy treatment decisions using genomics-based risk prediction estimates would improve cancer and quality of life outcomes.

**Methods:**

We developed a state-transition model to simulate outcomes over a 10 year horizon for a cohort of post-prostatectomy patients. Outcomes included cancer progression rates at 5 and 10 years, overall survival, and quality-adjusted survival with reductions for treatment, side effects, and cancer stage. We compared outcomes using population-level versus individual-level risk of cancer progression, and for genomics-based care versus usual care treatment recommendations.

**Results:**

Cancer progression outcomes, expected life-years (LYs), and expected quality-adjusted life-years (QALYs) were significantly different when individual genomics-based cancer progression risk estimates were used in place of population-level risk estimates. Use of the genomic classifier to guide treatment decisions provided small, but statistically significant, improvements in model outcomes. We observed an additional 0.03 LYs and 0.07 QALYs, a 12% relative increase in the 5-year recurrence-free survival probability, and a 4% relative reduction in the 5-year probability of metastatic disease or death.

**Conclusions:**

The use of genomics-based risk prediction to guide treatment decisions may improve outcomes for prostate cancer patients. This study offers a framework for individualized decision analysis, and can be extended to incorporate a wide range of personal attributes to enable delivery of patient-centered tools for informed decision-making.

## INTRODUCTION

Genomics-driven cancer medicine offers the potential for precision cancer treatment by delivering individualized information that patients and providers can use to make personalized medical decisions [1,2]. Recent advances in cancer genomics have created a myriad of genomic tests available for clinical use, but there has been little research done on how these genomic assays affect outcomes in cancer patients [3]. The clinical utility of these tests also needs to be demonstrated [3]. One way that genomic technologies may impact clinical decisions for localized cancer is by providing individual estimates of the risk of cancer progression or recurrence [4]. These individual estimates can then inform decisions regarding choices for more or less aggressive therapies [4].

Although randomized controlled trials demonstrate that patients with adverse pathological features post radical prostatectomy (RP) receiving adjuvant radiation therapy (ART) over observation have improved outcomes [5–9], the rates of ART utilization are low [10,11] and post-prostatectomy radiation therapy remains controversial [12,13]. Genomics-based risk stratification may address the issues of ART use following RP by creating choices of post-RP therapies that are based upon a patient’s individual predicted risk of metastasis. The ability to risk-stratify allows the clinician and patient to choose a post-RP therapy that is most effective for that individual. There are now several commercially available genomic assays that may stratify risk of prostate cancer aggressiveness [14]. A study of clinical decision-making for adjuvant and salvage therapies after RP has found genomic test results led urologists to change their treatment recommendations in more than 40% of case studies evaluated [15]. Although the impact of these tests on decision-making is substantial, the downstream long-term effects of genomic classifier (GC) risk-based precision medicine have not yet been evaluated. The current study aims to address this gap.

Comparative effectiveness research (CER) offers promise for evaluating new diagnostics tests for use in genomic and precision medicine [3,4] by generating evidence of the effectiveness of candidate genomic assays in a rapid and efficient manner. The individualized decision analysis methodology allows the models to represent the impact of individualized estimates of cancer progression upon population-level outcomes. This methodology emphasizes the particular importance of individual attributes on projected outcomes. The framework can be extended further to include a range of personal characteristics such as comorbid illnesses, tumor genotype, and individual utility estimates [16].

In this study, we developed a decision analytic approach to compare modeled outcomes—including expected time and quality-adjusted time over a ten-year horizon, and 5- and 10-year cancer progression rates—between scenarios with and without genomics-based risk. The goal of this approach was to estimate the potential clinical utility of a genomic test for informing clinical decisions. We use the Decipher GC test (GenomeDx Biosciences, San Diego, CA), a validated genomic assay that provides individual estimates of risk of metastasis (MET) after RP for high risk patients [17,18]. Our hypotheses are that: (1) individualized MET probabilities alter predicted outcomes, compared to average, population-level MET probabilities, for a substantial proportion of prostate cancer patients eligible for ART, and (2) the paradigm of genomics-driven cancer medicine improves predicted outcomes after RP when compared to decisions made in the absence of GC-based risk assessment.

## MATERIALS AND METHODS

**Fig 1:**
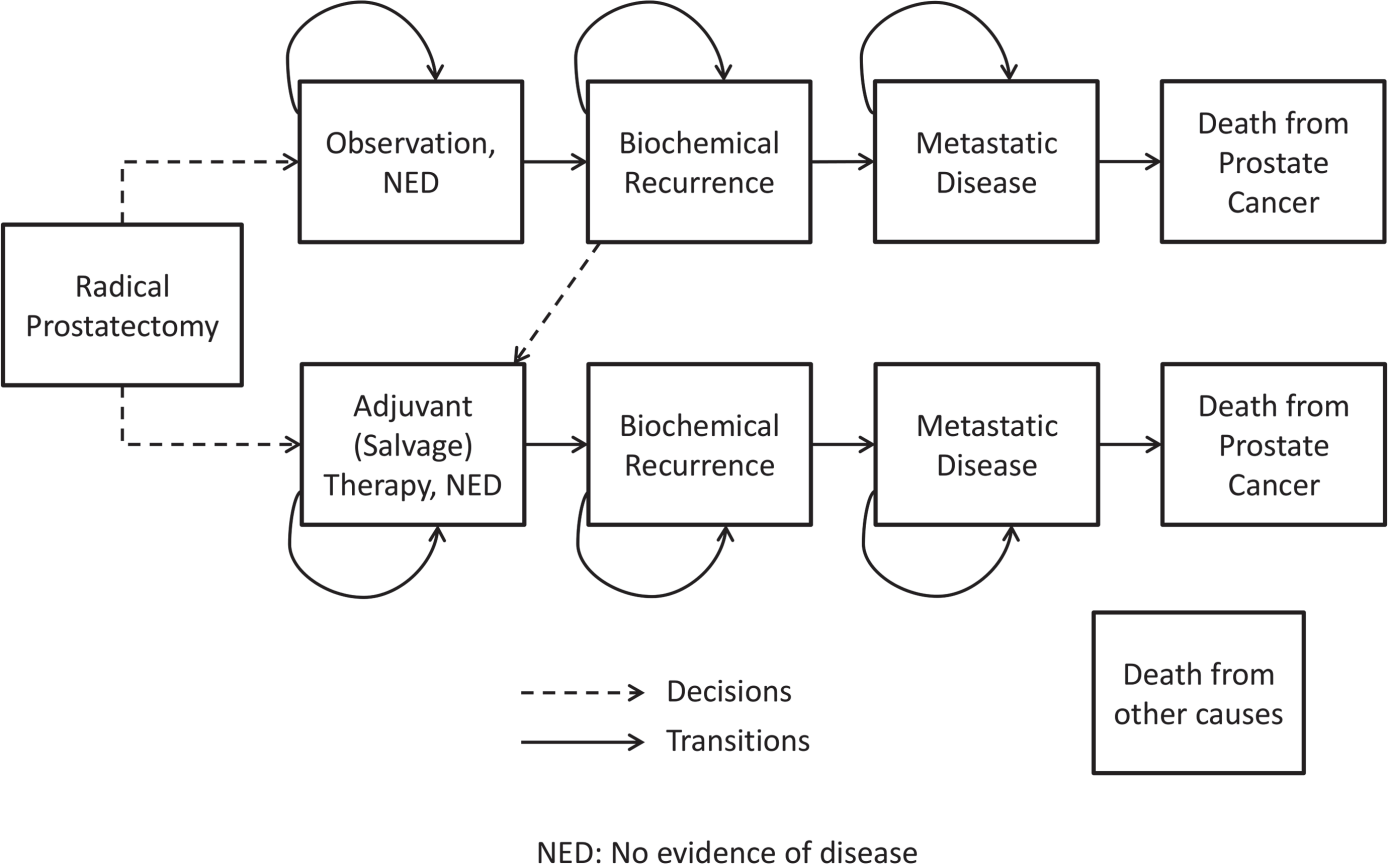
Simplified state transition diagram representing the treatment decisions and health state transitions post radical prostatectomy. NED represents patients with no evidence of disease.

We designed a state transition model [19] to estimate quality-adjusted life-expectancy for a cohort of men with prostate cancer who have received RP (Fig. 1). In the model, men are assigned to treatment with either early adjuvant therapy or close observation with salvage therapy for selected patients after biochemical recurrence (BCR). Treatment options used in the model are radiation therapy, hormone therapy, or both. Upon transitioning to the BCR or MET states, patients who have previously received treatment receive two years of hormone therapy. Individual subjects enter the model at the time of prostatectomy, and exit at death or the end of a 10-year horizon. The decision tree structure is depicted in S1 Fig. One-month cycle lengths were used. The Markov model state transitions are represented in Fig. 1. The model was coded in C/C++. The model validation is described in the Online Supporting Information files (see S1 Text).

A cohort simulation approach was applied, with two cohorts of RP patients used as the sampling populations. For each comparison in the experiments, we used the bootstrap method of sampling with replacement to choose each patient to pass through the model. We implemented paired sample runs to estimate the average difference in quality-adjusted life-years (QALYs) for each comparison. We chose a sample size of 10,000 to ensure estimation of expected QALYs to within 0.1 QALYs.

### Cohorts

Table 1 presents the two separate cohorts that were used for the experiments. The Mayo Clinic Cohort, originally reported for GC validation, consists of patients treated with RP between 2000 and 2006 at the Mayo Clinic that had one or more adverse pathology features [17]. The controls from the case-cohort study were reweighted [20] (with a weight of 5) to more accurately represent the severity mix of the US population of post-RP patients, yielding a simulation cohort of 808 patients with GC risk probabilities representing the risk of MET at five years. This reweighted cohort mirrors the Mayo Clinic Cohort validation study [17] performed for the GC and more accurately represents proportions of cases seen in the general population. The Thomas Jefferson University (TJU) Cohort consists of 139 patients with GC risk probabilities treated with RP and postoperative radiation between 1990 and 2009 at TJU Hospital [21]. For each patient in each cohort, the model uses: age at time of RP to define age-specific probability of non-cancer mortality, a 95% confidence interval for 5-year risk of metastasis, and risk status defined by GC as either lower or greater than six-percent average risk of metastasis, the threshold for differing treatment decisions based on the DECIDE study [15]. These individual-level data are available (S1 Data).

**Table 1:** Descriptions of patient characteristics for each cohort.

	**Mayo Clinic Cohort Ref. [17]** * **(n = 219)**	**TJU Cohort Ref. [21] (n = 139)**
**Age [Mean (SD)]**	63.28 (7.26)	60.71 (7.24)
**Gleason Score (%)**		
6	7	16
7	51	57
8–10	42	27
**Pathological Features (%)**		
ECE	43	82
SVI	37	38
PSM	56	75
LNI	13	0
**Adjuvant Therapies (%)**		
Hormone Therapy	34	9
Radiation Therapy	39	36
**Salvage Therapies (%)**		
Hormone Therapy	39	29
Radiation Therapy	31	64
**5 year Overall Cohort Metastatic Risk**	0.076	0.071
**Range of Risks for Cohort**	(0.0095, 0.48)	(0.0097, 0.32)
**Proportion Classified as High Risk According to GC**	41.46%	45.32%

SD = standard deviation; ECE = extracapsular extension; SVI = seminal vesicle invasion; PSM = positive surgical margin; LNI = lymph node involvement; GC = genomic classifier; TJU = Thomas Jefferson University; Ref. = reference

*Note, three patients were excluded from this original cohort due to unknown ECE status; Overall cohort metastatic risk, range of risks for cohort, and proportion classified as high risk according to GC was based on the reweighted cohort of 808 patients from the original cohort of 216 patients.

### Model Inputs

Sources for inputs were identified based upon a detailed review of a recently published decision analysis of ART versus salvage radiation therapy (SRT) [22] and a PubMed search using the terms “prostate cancer”, “radiation therapy”,”hormone therapy”, and “salvage” or “adjuvant”; and inclusion of published results from three randomized controlled trials of ART versus observation [5–8]. Probabilities of BCR and MET were extracted from the literature. Data regarding GC test performance characteristics was obtained from the published literature [15,17,18] (S1 Table). The model was designed with an assumption that the GC risk prediction was perfectly accurate for the sake of simplicity. However, it should be noted that the area under the receiver operating characteristic curve (AUC) for the GC test is 0.79 [17], suggesting some inherent uncertainty. It was also assumed that the therapeutic interventions had the same proportional effect on outcomes regardless of the magnitude of risk, given the lack of post-treatment clinical outcomes evidence stratified by GC-defined risk of metastasis. Probabilities for annual risk of death due to causes other than prostate cancer were based upon the most recent Centers for Disease Control and Prevention life tables [23], with adjustment for individual subjects based upon age at time of prostatectomy and time during the 10-year time horizon (S1 Table). Since the GC assay is new and data regarding how GC results influence real-world clinical practice are not yet available, the GC risk-dependent treatment decision probabilities were based upon the DECIDE study [15] (S2 Table). In the DECIDE study, GC-defined risk of metastasis higher than 6% was associated with higher rates of urologists recommending ART or SRT [15], so 6% was use as a threshold value for GC-based treatment decisions in the current model.

### Complications and Adverse Events

Treatment-dependent probabilities of BCR, MET, and death from prostate cancer as well as the probabilities of complications due to hormone therapy and radiation therapy are presented in S1 Table. These probabilities represent the probabilities of these events only due to these therapies and not to other treatments the patient may have received, including RP.

### Utilities

A utility is a value that is assigned based upon an individual patient’s preference for a particular health state, and potential values range from zero (death) to one (perfect health). Utility values obtained from the published literature represent the average preferences of healthy male patients who completed time trade-off experiments to determine how much time they would sacrifice to avoid a given health state [24]. The utilities associated with each complication and the health and treatment state utilities are presented in S1 Table. For patients in a particular health state that are also undergoing treatment and/or have incurred a complication, the patient’s utility is calculated by multiplying the utilities. QALYs are calculated by multiplying time (in years) by the utility; one QALY is equivalent to one year in perfect health or two years lived with a utility value of 0.5. We chose to use undiscounted QALYs since our analysis did not include costs [25].

### Sensitivity Analyses

One-way sensitivity analysis was performed to determine how sensitive results were to changes in key parameters. We individually varied transition probabilities, event probabilities, and utility estimates by ±10% to determine the impact on expected QALYs. Probabilistic sensitivity analysis was also performed on these parameters. Treatment decision probabilities, under usual care and those based on GC risk probabilities were varied to determine the effect on expected life-years (LYs), QALYs, and clinical outcomes. When varying the treatment probabilities, the relative probabilities of each type of treatment remained the same, but the percentage of patients on observation was changed to be 20% more or less than the base values in absolute terms with the maximum percentage on observation being 100% and the minimum being 0%.

### Statistical Comparisons

Paired sample simulations were generated for each comparison to minimize variation. Paired t-tests were used to determine if differences in expected LYs and QALYs were significant. We generated binomial proportion confidence intervals to compare clinical outcomes for each comparison. McNemar’s test was used to determine if differences in these outcomes were significant. Kaplan-Meier estimates for the overall probabilities of BCR and MET at 5 years were used for validation purposes.

## RESULTS

We developed a model with both population-level probabilities from the literature and individual-level probabilities based on the GC risk probability—the patient’s 5-year risk of metastasis. For the individual-level probabilities, monthly probabilities of BCR and MET were changed from the monthly population-level probabilities to reflect the patient’s individualized 5-year risk of metastasis. Changes were made based on the assumption that the individual’s monthly probabilities and the 5-year risk of metastasis are proportional to the population-level monthly probabilities and model-predicted 5-year risk of metastasis.

### Value of Incorporating Heterogeneity

We found that results differ for patient outcomes when individualized probabilities are used in place of population-level probabilities. For the Mayo Clinic Cohort, the population-level LYs were estimated to be 8.88 (95% CI: (8.84, 8.93)) and QALYs were estimated as 8.00 (95% CI: (7.95, 8.05)) compared to 8.82 LYs (95% CI: (8.77, 8.87)) and 8.03 QALYs (95% CI: (7.98, 8.08)) for the individualized model. Table 2 shows significant differences were observed for all of the 5- and 10-year BCR-free probabilities and the MET or death probabilities.

**Table 2:** Comparison of 5 and 10 year outcomes for population level probabilities vs. individual level probabilities using usual care treatment.

	**Population Level Probabilities**	**Individual Level Probabilities**	**P values**
**Mayo Clinic Cohort**			
5 year BCR free survival probability	0.572 (0.562, 0.581)	0.632 (0.623, 0.642)	< 0.001
10 year BCR free survival probability	0.321 (0.312, 0.330)	0.425 (0.416, 0.435)	< 0.001
5 year MET or Death probability	0.118 (0.111, 0.124)	0.135 (0.128, 0.142)	< 0.001
10 year MET or Death probability	0.302 (0.293, 0.311)	0.324 (0.315, 0.333)	< 0.001
**TJU Cohort**			
5 year BCR free survival probability	0.584 (0.574, 0.593)	0.624 (0.615, 0.633)	< 0.001
10 year BCR free survival probability	0.338 (0.329, 0.348)	0.415 (0.406, 0.425)	< 0.001
5 year MET or Death probability	0.098 (0.092, 0.103)	0.110 (0.104, 0.117)	< 0.001
10 year MET or Death probability	0.259 (0.250, 0.267)	0.276 (0.267, 0.285)	< 0.001

Results are presented for the Mayo Clinic and TJU cohorts.

For the TJU Cohort, the population-level LYs were estimated to be 9.10 (95% CI: (9.05, 9.14)) and QALYs were estimated as 8.18 (95% CI: (8.13, 8.22)) compared to 9.04 LYs (95% CI: (8.99, 9.08)) and 8.19 QALYs (95% CI: (8.15, 8.24)) for the individualized model. As seen in Table 2, significant differences were found for the 5- and 10-year BCR-free probabilities and the MET or death probabilities.

The population-level version of the model estimates greater expected LYs and slightly lower QALYs compared to when individualized probabilities are used. These differences and the differences in BCR and MET outcomes occur despite the fact that both versions of the model incorporate age-specific estimates of probability of death from other causes.

### Influence of Genomics-Based Decisions

We used the heterogeneous version of the model to compare the use of genomics-based treatment decisions to usual care treatment decisions. For the Mayo Clinic Cohort, GC-based treatment resulted in greater expected LYs—8.85 vs. 8.82 (p<0.001)—and greater expected QALYs—8.10 vs. 8.03 (p<0.001). For the TJU Cohort, GC-based treatment resulted in greater expected LYs—9.07 vs. 9.04 (p<0.001)—and greater expected QALYs—8.25 vs. 8.19 (p < 0.001). In addition, from Table 3 we see that the 5- and 10-year outcomes for BCR and MET are significantly better under GC-based treatment with the exception of the 5-year probability of MET or death for the Mayo Clinic Cohort.

**Table 3:** Comparison of 5 and 10 year outcomes for usual care vs. genomics-based care decisions using individual level probabilities.

	**Usual Care Treatment**	**GC-Based Treatment**	**P values**
**Mayo Clinic Cohort**			
5 year BCR free survival probability	0.632 (0.623, 0.642)	0.707 (0.698, 0.716)	< 0.001
10 year BCR free survival probability	0.425 (0.416, 0.435)	0.496 (0.486, 0.505)	< 0.001
5 year MET or Death probability	0.135 (0.128, 0.142)	0.129 (0.122, 0.135)	0.115
10 year MET or Death probability	0.324 (0.315, 0.333)	0.307 (0.298, 0.316)	< 0.001
**TJU Cohort**			
5 year BCR free survival probability	0.624 (0.615, 0.633)	0.705 (0.696, 0.714)	< 0.001
10 year BCR free survival probability	0.415 (0.406, 0.425)	0.495 (0.485, 0.505)	< 0.001
5 year MET or Death probability	0.110 (0.104, 0.117)	0.106 (0.100, 0.112)	0.007
10 year MET or Death probability	0.276 (0.267, 0.285)	0.261 (0.252, 0.269)	< 0.001

Results are presented for the Mayo Clinic and TJU cohorts. McNemar’s test was used to test for significant differences between usual care and GC-based treatment outcomes for each cohort.

BCR = biochemical recurrence; MET = metastasis; GC = genomic classifier.

**Fig 2:**
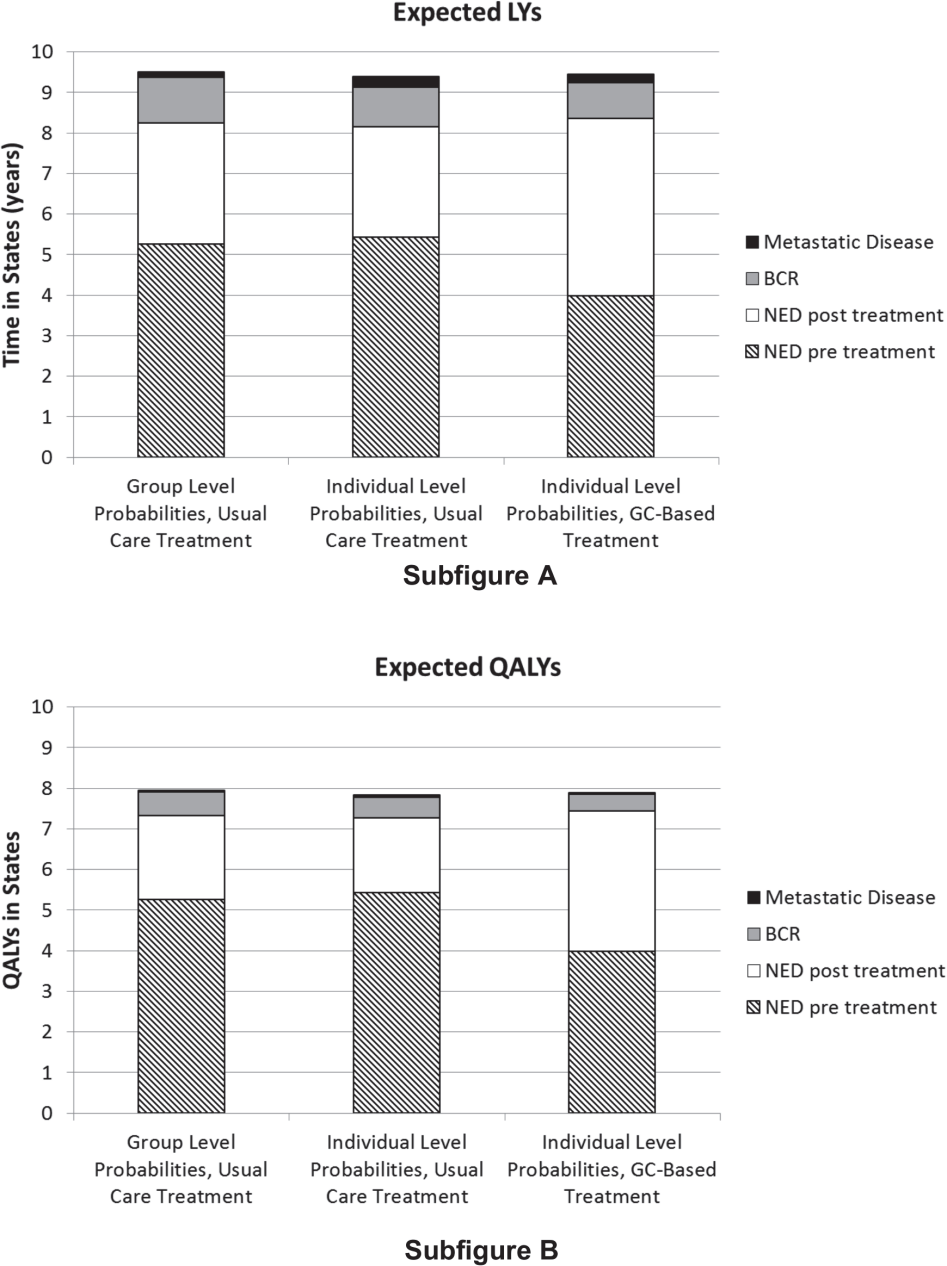
Time in life years (LYs) in states (Subfigure A), and quality-adjusted life years (QALYs) in states (Subfigure B) for the Mayo Clinic Cohort. GC-based treatment refers to treatment decisions made based upon the genomic risk classifier assay. BCR = biochemical recurrence; NED = no evidence of disease; GC = genomic classifier.

We also observe from Fig. 2 that while treatment occurs earlier on average (due to a larger percentage of patients receiving adjuvant therapy) BCR occurs later under GC-based treatment than usual care treatment when considering LYs and QALYs. The same is true for the TJU Cohort results shown in S2 Fig. As a result, patients under GC-based treatment spent more time in the no evidence of disease (NED) clinical state. For the Mayo Clinic Cohort, the average time spent in NED under genomics-based care is 7.74 LYs compared to 7.59 LYs under usual care (p<0.001); for the TJU Cohort the average time spent in NED under genomics-based care is 7.84 LYs compared to 7.70 LYs under usual care (p<0.001). Sensitivity analysis results are presented in S3 Fig. and S3 Table.

## DISCUSSION

We developed a decision analytic model designed to evaluate the influence of individualized probabilities of metastasis after RP on outcomes. We observed that incorporating individual GC risk as inputs for risk of developing MET, compared to population-level values, resulted in different outcomes. This suggests that consideration of individualized inputs for disease progression may refine estimates developed using decision analytic methodologies. The use of GC risk to guide treatment decisions was associated with small, but significant, increases in LYs, QALYs, and time spent in the NED pre- and post-treatment states, as well as improved 5- and 10-year outcomes. To our knowledge, this is the first published report to use individualized decision analysis methodology to evaluate the clinical utility of a genomics-based risk stratification assay in prostate cancer. This CER approach provides a potential model for estimating the clinical utility of any genomic assay when prospective clinical outcomes data are not yet available.

These findings suggest that considering individual GC risk probabilities after prostatectomy may improve clinical outcomes for prostate cancer patients. Clinical significance is primarily seen from the statistically significant improvements in 5- and 10-year probabilities of BCR, MET, and death. In addition, the magnitude of benefit in QALYs observed in the current study is similar to those reported for genomic prognostic assays designed to inform adjuvant chemotherapy decisions for breast and colon cancer, including the OncotypeDX Breast Cancer Test (Genomic Health Inc., Redwood City, CA) that is currently in clinical use [26–28]. Sensitivity analysis suggests that more aggressive use of salvage and adjuvant therapies for high GC scores could expand the observed increases in QALYs by up to 0.08 QALYs. This provides further evidence that the use of genomic risk stratification assays to guide treatment decisions can improve outcomes.

This study contributes to the evidence base for genomics-driven prostate cancer care by estimating the potential downstream clinical utility of a genomic assay designed to provide individualized predictions of the risk of metastasis. Prior reports demonstrated the importance of considering individual patient preferences for treatment outcomes when comparing prostate cancer treatments using decision analytic methodology [16,29,30]. In the current study, we extended the individualized decision analysis framework to include personalized GC risk results as personalized inputs in a decision analysis of adjuvant and salvage therapies for prostate cancer. This methodology can be a useful approach for representing heterogeneity among patients in the form of genomics-defined probabilities of treatment outcomes, and the heuristic model can be extended to include a broad range of personal attributes in addition to tumor genomics.

Decision analysis models are limited by the quality and accuracy of model inputs. The utility inputs used in the current model were obtained from the published literature and were incorporated in the model as population-level averages. Previously it has been shown in prostate cancer that population-level utility estimates result in decision analysis recommendations that differ from those using individualized utility values [16,29,30]. This was beyond the scope of the current study, and inclusion of personalized utility estimates would have presented a separate list of methodological challenges [31]. In addition, the improvements in observed outcomes in the current study must be weighed against the additional costs of providing genomics-driven cancer care. A detailed cost-effectiveness analysis of the GC assay is beyond the scope of this report, but is the subject of ongoing research by our group.

Currently, level I evidence is not available regarding the relative effectiveness of adjuvant and salvage prostate cancer treatment among patients with high versus low GC risk probabilities but this data could refine estimates of treatment success and further influence predicted outcomes. In essence, the GC assay reclassifies individual patients based upon risk of metastatic disease, providing individualized information that can guide therapeutic decisions. In doing so, the GC assay may provide a basis to streamline and refine decision-making for ART and SRT for prostate cancer patients, an area of considerable controversy and uncertainty in contemporary clinical practice [32–34]. Whether this will lead to downstream improvement in prostate cancer death rates will relate in part to the availability of effective therapies to prevent metastasis. However, the GC assay may at the very least provide a quantitative tool for identifying those patients with low risk of metastasis who may be spared the toxicity of ART. One limitation of the current model is the assumption that adjuvant and salvage therapies have similar proportional effectiveness regardless of GC risk. The relative effectiveness of adjuvant and salvage treatments for higher and lower risk patients would be expected to also influence the model outcomes. We addressed this limitation by subjecting the effectiveness of adjuvant and salvage therapy (i.e., probability of redistribution to the NED state) and the likelihood of receiving treatment based upon GC score to sensitivity analyses. Our model also assumed perfect performance of the GC test, despite some inherent uncertainty in the assay’s prediction of the probability of metastasis at 5 years after RP [17]. It is unlikely that this influenced the model results, which were robust to a range of one-way sensitivity analyses as well as probabilistic sensitivity analysis.

Our findings suggest that genomic risk probabilities offer statistically significant clinical benefit for prostate cancer patients faced with decisions for second-line therapy after prostatectomy. This is shown through the improvements observed in LYs, QALYs, and probabilities of BCR, MET, and death when treatment decisions were made based upon GC risk rather than usual care. Our application of individualized decision analysis demonstrates the usefulness of CER for estimating clinical utility of genomics-based risk prediction assays. Future applications of individualized decision analytic methods for precision medicine diagnostics should include the following: evaluation of cost-effectiveness; incorporation of additional subject-level attributes including other clinical and pathological factors, medical comorbidities, and genomics-based predictors of normal tissue injury or treatment response; and patient preferences for treatment options. These additional considerations can create a decision analysis framework that represents a comprehensive platform for personalized medicine.

## Supporting Information

S1 Text Validation and Sensitivity Analysis. This section includes full details regarding sensitivity analysis.
(DOCX)

S1 Data Individual-level data for patient age and for genomic classifier-predicted 5-year probability of metastasis for cohorts evaluated in this model. These data comprise the individualized inputs for the decision analytic model.
(XLS)

S1 Fig Decision tree structure for the post radical prosatectomy decision making process. The salvage and radiation therapy arms (labeled as Salvage Tx and Adjuvant Tx) each represent the treatments radiation therapy, hormone therapy, and radiation and hormone therapy.
(TIFF)

S2 Fig Time in life years (LYs) in states (Subfigure A), and quality-adjusted life years (QALYs) in states (Subfigure B) for the TJU Cohort. BCR = biochemical recurrence; NED = no evidence of disease; TJU = Thomas Jefferson University; GC = genomic classifier.
(TIFF)

S3 Fig Effects on QALYs from changes in probability values by +/-10% for the Mayo Clinic Cohort. GC-based treatment refers to treatment decisions made based upon the genomic risk classifier assay. BCR = biochemical recurrence; GC = genomic classifier.
(TIFF)

S1 Table Transition probability and utility inputs used as group-level inputs for the model. When patients are on treatment, the health state utility is multiplied by the treatment state utility. Citations are included [5, 8, 22–24, 37–42], as well as notes to explain when the probabilities and utilities are applied within the model. Unless otherwise states, the range used in the sensitivity analysis was 10% higher and lower than the input value.
(DOCX)

S2 Table Treatment decision probabilities for adjuvant and salvage treatments. The standard of care probabilities are derived from the published literature [10,11,35–37], and reflect likelihood of receiving treatment under usual care settings. The alternative, genomic-classifier score-based treatment probabilities were derived from a published report to assess physician recommendations for treatment based upon the genomic classifier test. For treatment decisions, genomic classifier risk scores were considered either low-risk or high-risk based upon a threshold estimated risk of distant metastasis of 6% at 5 years, consistent with the observed influence of genomic classifier risk scores on urologists’ recommendations in the DECIDE study [15]. For sensitivity analyses, a range of +/- 20% was used for standard of care observation decisions to evaluate inaccuracies in baseline estimates; a range of +/- 20% was used for genomic classifier risk score-based observation treatment probabilities to model the effects of more or less aggressive incorporation of genomic risk data into clinical practice. *Standard of care therapy utilization rates were selected empirically based upon a combination of expert opinion and published reports of radiation therapy utilization after radical prostatectomy [10,11, 35–37]. GC = genomic classifier.
(DOCX)

S3 Table Sensitivity analysis results for the Mayo Clinic Cohort when varying treatment recommendation aggressiveness. GC = genomic classifier.
(DOCX)
