# Supplementary material for: Evaluating the Clinical Impact of a Genomic Classifier in Prostate Cancer Using Individualized Decision Analysis
Source: PLoS One. 2015 Apr 2;10(4):e0116866. doi: 10.1371/journal.pone.0116866 (PMC4383561; doi:10.1371/journal.pone.0116866)
Supplement: S1 Text — This section includes full details regarding sensitivity analysis. (DOCX) [file pone.0116866.s001.docx]

**SUPPORTING INFORMATION**

**Text S1 Section: Validation and Sensitivity Analysis.** This section includes full details regarding sensitivity analysis.

**Validation**

The model results generated using individualized parameters were validated against 5-year estimates from the cohort validation study [17] using the treatment decision probabilities that each cohort received. The Mayo Clinic Cohort 5-year BCR-free survival probability was 0.662 (95% CI: (0.628, 0.698)) compared to the model-predicted 0.672 (95% CI: (0.666, 0.684)), and the 5-year probability of MET or death was 0.070 (95% CI: (0.051, 0.089)) compared to the model-predicted 0.129 (95% CI: (0.122, 0.135)). The TJU Cohort 5-year BCR-free survival probability was 0.649 (95% CI: (0.566, 0.745)) compared to the model-predicted 0.660 (95% CI: (0.651, 0.669)), and the 5-year probability of MET or death was 0.056 (95% CI: (0.011, 0.099)) compared to the model-predicted 0.103 (95% CI: (0.097, 0.109)). The 5-year BCR-free survival probabilities were nearly identical to observed probabilities. The model-predicted values for the 5-year MET probabilities were mainly influenced by the rate of death from other causes. It is possible that the Mayo Clinic Cohort and the TJU Cohort were slightly healthier populations than the general populations, accounting for these differences.

**Sensitivity Analysis**

Our model findings are robust with respect to changes in the model parameters. Figure S3 shows the effect on QALYs when the probability parameters are changed by ±10%. Overall, the genomics-based care QALYs are less sensitive to changes in probabilities than the usual-care QALYs. Changing the probability of BCR produces the largest change in QALYs of all of the probability parameters, with an uncertainty range of 0.17 QALYs for usual care versus 0.13 QALYs for genomics-based care. Changing the probability of death from other causes produces large changes in QALYs as well. GC-based treatment is more robust to changes in BCR probabilities than usual care treatment. Among the changes in utility values, changing the utility values for clinical states has the largest effect on expected QALYs, over changes in values for treatment and side effects. This change in QALYs due to changing utility values for clinical states is primarily due to the 10% reduction in the utility value for being in the NED state. While the QALY values are sensitive to changes in the utility value used for NED, the difference in QALYs between usual care and GC-based care is comparable to the difference QALYs with the utility value of 1.0 for NED.

We also tested the sensitivity of the results with respect to the aggressiveness of the treatment recommendations. We modeled less aggressive and more aggressive treatments with a 20% absolute increase and a 20% absolute decrease, respectively, by changing the percentage observed for the adjuvant and salvage therapy decisions. When each set of guidelines was less aggressive, the expected LYs were 0.05 LYs greater while the expected QALYs were 0.09 QALYs greater under GC-based care, though neither were statistically different. When each set of guidelines was more aggressive, the expected LYs were not statistically different and expected QALYs were greater by 0.06 QALYs using GC-based treatment, though not statistically different. In addition, the expected 5- and 10-year metastatic disease outcomes were in general more sensitive to changes in usual care treatment recommendations than GC-based treatment recommendations. The complete results from the treatment recommendations sensitivity analysis experiments can be found in Table S3.

Probabilistic sensitivity analysis resulted in greater LYs for GC-based care – 8.89 vs. 8.86 (p < 0.001) – and greater QALYs for GC-based care – 6.91 vs. 6.85 (p < 0.001). The GC-based care was more favorable than usual care in terms of the 5-year BCR-free probabilities – 0.712 vs. 0.638 (p < 0.001), 10-year BCR-free probabilities – 0.498 vs. 0.426 (p < 0.001), 5-year probabilities of MET or death – 0.123 vs. 0.130 (p = 0.051), and 10-year probabilities of MET or death – 0.296 vs. 0.315 (p < 0.001). The results of the probabilistic sensitivity analysis show that the model findings are robust.
