# Supplementary figures and images for: Evaluating the Clinical Impact of a Genomic Classifier in Prostate Cancer Using Individualized Decision Analysis
Source: PLoS One. 2015 Apr 2;10(4):e0116866. doi: 10.1371/journal.pone.0116866 (PMC4383561; doi:10.1371/journal.pone.0116866)

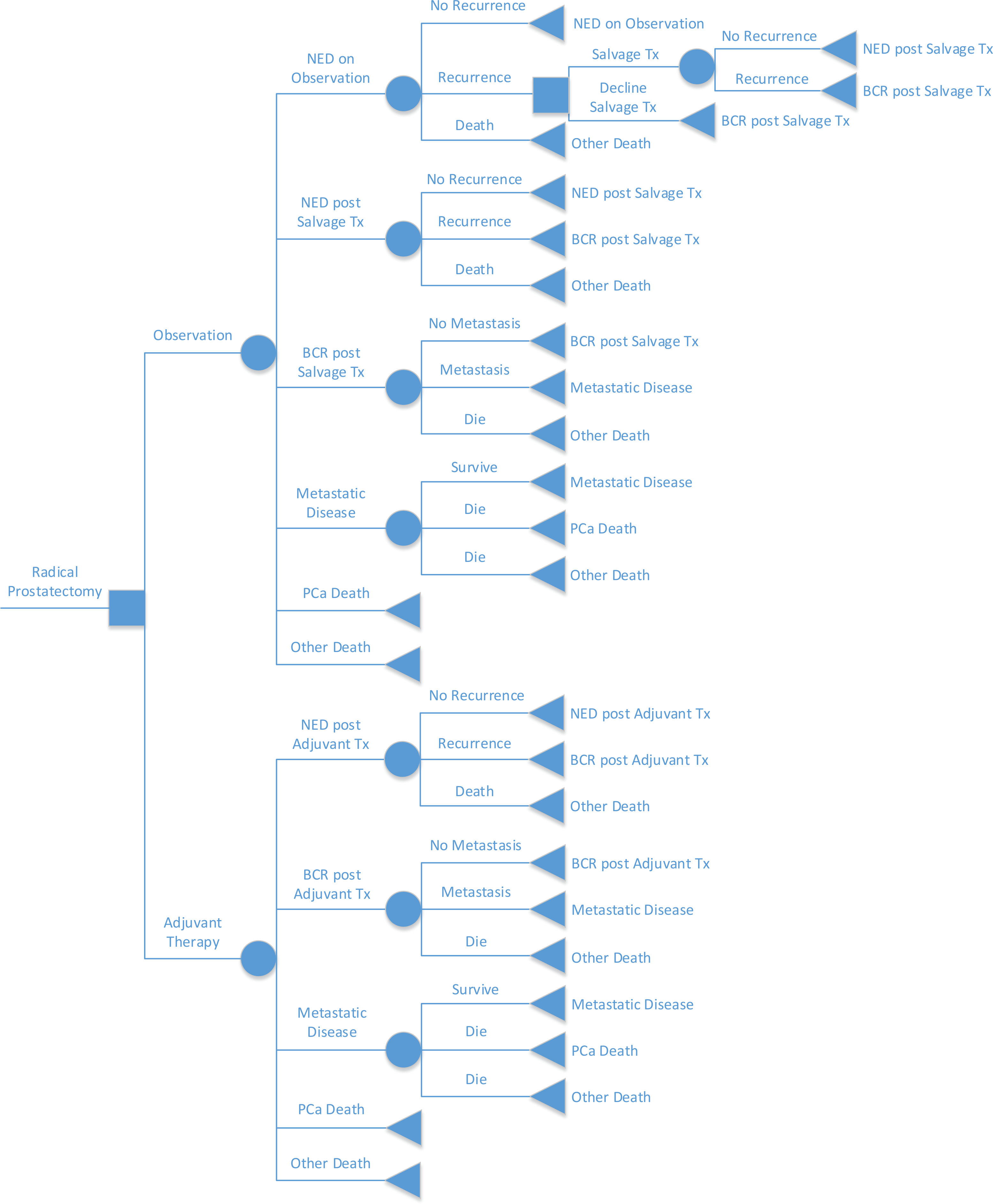

Supplement: S1 Fig — The salvage and radiation therapy arms (labeled as Salvage Tx and Adjuvant Tx) each represent the treatments radiation therapy, hormone therapy, and radiation and hormone therapy. (TIFF) [file pone.0116866.s003.tiff]

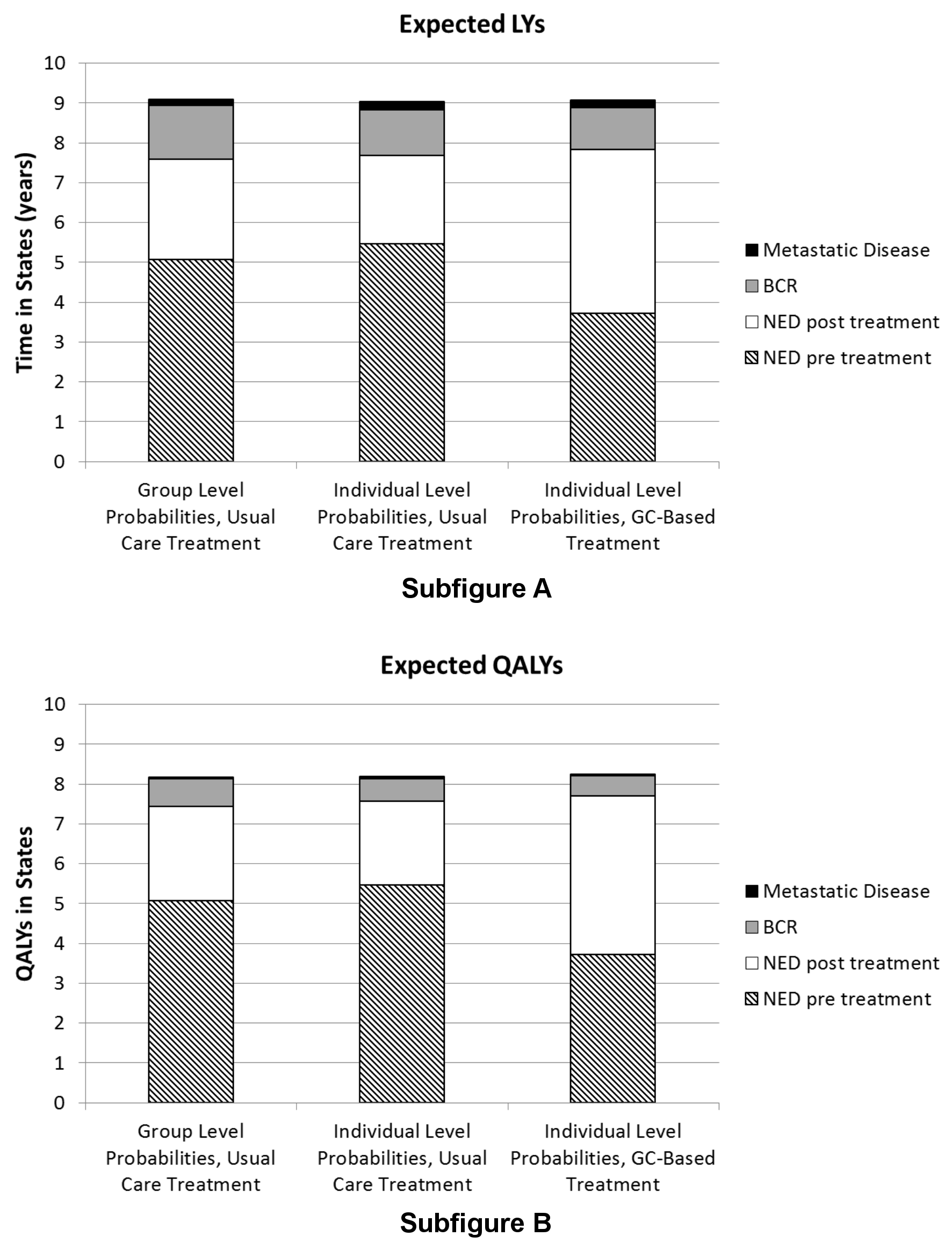

Supplement: S2 Fig — BCR = biochemical recurrence; NED = no evidence of disease; TJU = Thomas Jefferson University; GC = genomic classifier. (TIFF) [file pone.0116866.s004.tiff]

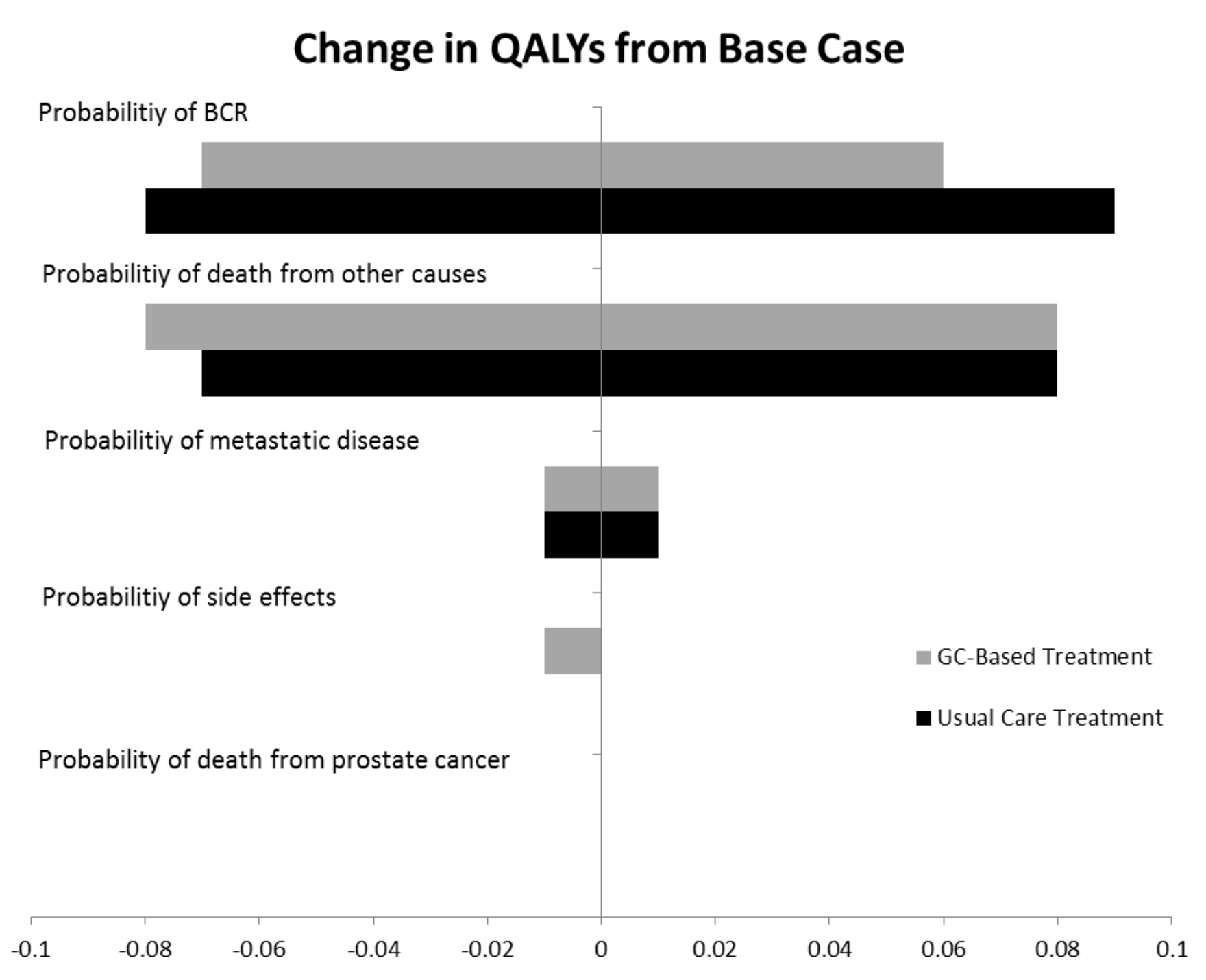

Supplement: S3 Fig — GC-based treatment refers to treatment decisions made based upon the genomic risk classifier assay. BCR = biochemical recurrence; GC = genomic classifier. (TIFF) [file pone.0116866.s005.tiff]
