# Supplementary material for: Evaluating the Clinical Impact of a Genomic Classifier in Prostate Cancer Using Individualized Decision Analysis
Source: PLoS One. 2015 Apr 2;10(4):e0116866. doi: 10.1371/journal.pone.0116866 (PMC4383561; doi:10.1371/journal.pone.0116866)
Supplement: S1 Table — When patients are on treatment, the health state utility is multiplied by the treatment state utility. Citations are included [5, 8, 22–24, 37–42], as well as notes to explain when the probabilities and utilities are applied within the model. Unless otherwise states, the range used in the sensitivity analysis was 10% higher and lower than the input value. (DOCX) [file pone.0116866.s006.docx]

**Table S1: Transition probability and utility inputs.** Transition probability and utility inputs used as group-level inputs for the model. When patients are on treatment, the health state utility is multiplied by the treatment state utility. Citations are included [5, 8, 22-24, 37-42], as well as notes to explain when the probabilities and utilities are applied within the model. Unless otherwise states, the range used in the sensitivity analysis was 10% higher and lower than the input value.

|  | **Input Value^*^** | **Sensitivity Analysis Range^#^** | **Citations/Notes** |
| --- | --- | --- | --- |
| **Disease Progression Probabilities (annual)** | | | |
| **BCR from NED state** |  |  |  |
| Under Observation | 0.0945 | +/- 10% | Wiegel et al. [8] |
| After Salvage Therapy | 0.1416 | +/- 10% | Stephenson et al. [38] |
| After Adjuvant Therapy | 0.039 | +/- 10% | Thompson et al. [5] |
| **Metastasis from BCR state** |  |  |  |
| Under Observation | 0.065 | +/- 10% | Wiegel et al. [8] |
| After Salvage Therapy | 0.065 | +/- 10% | Wiegel et al. [8] |
| After Adjuvant Therapy | 0.057 | +/- 10% | Wiegel et al. [8] |
| **Prostate Cancer Death from Metastasis state** |  |  |  |
| Under Observation | 0.2412 | +/- 10% | Boorjian et al. [39] |
| After Salvage Therapy | 0.2412 | +/- 10% | Boorjian et al. [39] |
| After Adjuvant Therapy | 0.2412 | +/- 10% | Boorjian et al. [39] |
| **Complication Probabilities after Treatments** | | | |
| **Fracture** | 0.014 | +/- 10% | Krupski et al. [40]  Occurs in patients who have had hormone therapy. |
| **Erectile Dysfunction** | 0.04 | +/- 10% | Elliott et al. [22]  Occurs in patients who have had radiation therapy. |
| **Urinary Obstruction** | 0.12 | +/- 10% | Elliott et al. [22]  Occurs in patients who have had radiation therapy |
| **Urinary Incontinence** | 0.04 | +/- 10% | Elliott et al. [22]  Occurs in patients who have had radiation therapy |
| **Bowel Dysfunction** | 0.18 | +/- 10% | Elliott et al. [22]  Occurs in patients who have had radiation therapy |
| **Probabilities for Annual Non-Cancer Mortality Risk** | | | |
| **By Age Group (years):** |  |  |  |
| 40-44 | 0.002484 | +/- 10% | National Vital Statistics Report [23] |
| 45-49 | 0.003999 | +/- 10% | [23] |
| 50-54 | 0.006124 | +/- 10% | [23] |
| 55-59 | 0.009042 | +/- 10% | [23] |
| 60-64 | 0.012622 | +/- 10% | [23] |
| 65-69 | 0.018443 | +/- 10% | [23] |
| 70-74 | 0.028049 | +/- 10% | [23] |
| 75-79 | 0.044161 | +/- 10% | [23] |
| 80-84 | 0.072806 | +/- 10% | [23] |
| 85+ | 0.152416 | +/- 10% | [23] |
| **Utility Values for Health of Treatment State** | | | |
| **NED** | 1 | -10% | Assumption |
| **Death** | 0 | n/a | Assumption |
| **BCR (no salvage/adjuvant therapy)** | 0.68 | +/- 10% | Hayes et al. [41] |
| **BCR post treatment failure** | 0.49 | +/- 10% | Stewart et al. [24] |
| **Metastasis** | 0.25 | +/- 10% | Stewart et al. [24] |
| **Radiation Therapy** | 0.73 | +/- 10% | Stewart et al. [24]  During a 3-month interval. |
| **Hormone Therapy** | 0.73 | +/- 10% | Konski et al. [37] |
| **Complication-Related Utility Inputs** | | | |
| **Fracture** | 0.83 first year,  0.87 thereafter | +/- 10% | Schousboe et al. [42] |
| **Erectile Dysfunction** | 0.89 | +/- 10% | Stewart et al. [24] |
| **Urinary Obstruction** | 0.88 | +/- 10% | Stewart et al. [24] |
| **Urinary Incontinence** | 0.83 | +/- 10% | Stewart et al. [24], 5 years only. |
| **Bowel Dysfunction** | 0.71 | +/- 10% | Stewart et al. [24], 2 years only. |

BCR = biochemical recurrence; NED = no evidence of disease. The following citations are referenced in Table S1 and are not listed in the main text.
