# Supplementary material for: Evaluating the Clinical Impact of a Genomic Classifier in Prostate Cancer Using Individualized Decision Analysis
Source: PLoS One. 2015 Apr 2;10(4):e0116866. doi: 10.1371/journal.pone.0116866 (PMC4383561; doi:10.1371/journal.pone.0116866)
Supplement: S2 Table — The standard of care probabilities are derived from the published literature [10,11,35–37], and reflect likelihood of receiving treatment under usual care settings. The alternative, genomic-classifier score-based treatment probabilities were derived from a published report to assess physician recommendations for treatment based upon the genomic classifier test. For treatment decisions, genomic classifier risk scores were considered either low-risk or high-risk based upon a threshold estimated risk of distant metastasis of 6% at 5 years, consistent with the observed influence of genomic classifier risk scores on urologists’ recommendations in the DECIDE study [15]. For sensitivity analyses, a range of +/- 20% was used for standard of care observation decisions to evaluate inaccuracies in baseline estimates; a range of +/- 20% was used for genomic classifier risk score-based observation treatment probabilities to model the effects of more or less aggressive incorporation of genomic risk data into clinical practice. *Standard of care therapy utilization rates were selected empirically based upon a combination of expert opinion and published reports of radiation therapy utilization after radical prostatectomy [10,11, 35–37]. GC = genomic classifier. (DOCX) [file pone.0116866.s007.docx]

|  |  | **Using GC Risk Score**  **[based upon Badani et al., 2013 [15]]** | |
| --- | --- | --- | --- |
|  | **Standard of Care *** | **Low Risk Patients** | **High Risk Patients** |
| **Adjuvant Therapy** | | | |
| Observation | 85.8% | 80% | 18% |
| Radiation Therapy | 7.2% | 19% | 55% |
| Hormone Therapy | 3% | 1% | 2% |
| Radiation Therapy + Hormone Therapy | 4% | 0% | 25% |
| **Salvage Therapy** | | | |
| Observation | 20.5% | 20% | 20% |
| Radiation Therapy | 45.6% | 60% | 35% |
| Hormone Therapy | 3.3% | 0% | 5% |
| Radiation Therapy + Hormone Therapy | 30.6% | 20% | 40% |

**Table S2: Treatment decision probabilities.** Treatment decision probabilities for adjuvant and salvage treatments. The standard of care probabilities are derived from the published literature [10,11, 35-37], and reflect likelihood of receiving treatment under usual care settings. The alternative, genomic-classifier score-based treatment probabilities were derived from a published report to assess physician recommendations for treatment based upon the genomic classifier test. For treatment decisions, genomic classifier risk scores were considered either low-risk or high-risk based upon a threshold estimated risk of distant metastasis of 6% at 5 years, consistent with the observed influence of genomic classifier risk scores on urologists’ recommendations in the DECIDE study [15]. For sensitivity analyses, a range of +/- 20% was used for standard of care observation decisions to evaluate inaccuracies in baseline estimates; a range of +/- 20% was used for genomic classifier risk score-based observation treatment probabilities to model the effects of more or less aggressive incorporation of genomic risk data into clinical practice.

**Standard of care therapy utilization rates were selected empirically based upon a combination of expert opinion and published reports of radiation therapy utilization after radical prostatectomy [10,11,* 35-37*]. GC = genomic classifier.*
