# Supplementary material for: Evaluating the Clinical Impact of a Genomic Classifier in Prostate Cancer Using Individualized Decision Analysis
Source: PLoS One. 2015 Apr 2;10(4):e0116866. doi: 10.1371/journal.pone.0116866 (PMC4383561; doi:10.1371/journal.pone.0116866)
Supplement: S3 Table — GC = genomic classifier. (DOCX) [file pone.0116866.s008.docx]

|  | **Usual Care Treatment** | **GC-Based Treatment** |
| --- | --- | --- |
| **Less Aggressive** |  |  |
| 5 year BCR free survival probability | 0.611 (0.601, 0.620) | 0.675 (0.666, 0.685) |
| 10 year BCR free survival probability | 0.402 (0.392, 0.412) | 0.462 (0.452, 0.471) |
| 5 year MET or Death probability | 0.145 (0.138, 0.152) | 0.134 (0.128, 0.141) |
| 10 year MET or Death probability | 0.339 (0.330, 0.348) | 0.318 (0.309, 0.327) |
| LYs | 8.77 (8.73, 8.82) | 8.82 (8.78, 8.87) |
| QALYs | 7.97 (7.92, 8.03) | 8.06 (8.01, 8.11) |
| **Base Case** |  |  |
| 5 year BCR free survival probability | 0.632 (0.623, 0.642) | 0.707 (0.698, 0.716) |
| 10 year BCR free survival probability | 0.425 (0.416, 0.435) | 0.496 (0.486, 0.505) |
| 5 year MET or Death probability | 0.135 (0.128, 0.142) | 0.129 (0.122, 0.135) |
| 10 year MET or Death probability | 0.324 (0.315, 0.333) | 0.307 (0.298, 0.316) |
| LYs | 8.82 (8.77, 8.87) | 8.85 (8.81, 8.90) |
| QALYs | 8.03 (7.98, 8.08) | 8.10 (8.05, 8.15) |
| **More Aggressive** |  |  |
| 5 year BCR free survival probability | 0.662 (0.653, 0.672) | 0.732 (0.724, 0.741) |
| 10 year BCR free survival probability | 0.458 (0.448, 0.468) | 0.525 (0.515, 0.535) |
| 5 year MET or Death probability | 0.126 (0.120, 0.133) | 0.126 (0.120, 0.133) |
| 10 year MET or Death probability | 0.311 (0.302, 0.320) | 0.302 (0.293, 0.311) |
| LYs | 8.86 (8.81, 8.91) | 8.86 (8.82, 8.91) |
| QALYs | 8.05 (8.00, 8.10) | 8.11 (8.06, 8.16) |

Table S3: Treatment Recommendation Sensitivity Analysis. Sensitivity analysis results for the Mayo Clinic Cohort when varying treatment recommendation aggressiveness. GC = genomic classifier.
